# “I have to listen to them or they might harm me” and other narratives of why women endure obstetric violence in Bihar, India

**DOI:** 10.1111/birt.12828

**Published:** 2024-06-05

**Authors:** Kaveri Mayra, Zoë Matthews, Jane Sandall, Sabu S. Padmadas

**Affiliations:** ^1^ Birth Place Lab, Department of Family Practice, Faculty of Medicine University of British Columbia Vancouver British Columbia Canada; ^2^ Department of Social Statistics and Demography, Faculty of Social Sciences University of Southampton Southampton UK; ^3^ Department of Women and Children's Health, School of Life Course Sciences Kings College London London UK

**Keywords:** gender‐based violence, maternal health, obstetric violence, perinatal care, respectful maternity care

## Abstract

**Background:**

Evidence suggests that obstetric violence has been prevalent globally and is finally getting some attention through research. This human rights violation takes several forms and is best understood through the narratives of embodied experiences of disrespect and abuse from women and other people who give birth, which is of utmost importance to make efforts in implementing respectful maternity care for a positive birthing experience. This study focused on the drivers of obstetric violence during labor and birth in Bihar, India.

**Methods:**

Participatory qualitative visual arts‐based method of data collection—body mapping‐assisted interviews (adapted as birth mapping)—was conducted to understand women's perception of why they are denied respectful maternity care and what makes them vulnerable to obstetric violence during labor and childbirth. This study is embedded in feminist and critical theories that ensure women's narratives are at the center, which was further ensured by the feminist relational discourse analysis. Eight women participated from urban slums and rural villages in Bihar, for 2–4 interactions each, within a week. The data included transcripts, audio files, body maps, birthing stories, and body key, which were analyzed with the help of NVivo 12.

**Findings:**

Women's narratives suggested drivers that determine how they will be treated during labor and birth, or any form of sexual, reproductive, and maternal healthcare seeking presented through the four themes: (1) “I am admitted under your care, so, I will have to do what you say”—Influence of power on care during childbirth; (2) “I was blindfolded … because there were men”—Influence of gender on care during childbirth; (3) “The more money we give the more convenience we get”—Influence of structure on care during childbirth; and (4) “How could I ask him, how it will come out?”—Influence of culture on care during childbirth. How women will be treated in the society and in the obstetric environment is determined by their identity at the intersections of age, class, caste, marital status, religion, education, and many other sociodemographic factors. The issues related to each of these are intertwined and cross‐cutting, which made it difficult to draw clear categorizations because the four themes influenced and overlapped with each other. Son preference, for example, is a gender‐based issue that is part of certain cultures in a patriarchal structure as a result of power‐based imbalance, which makes the women vulnerable to disrespect and abuse when their baby is assigned female at birth.

**Discussion:**

Sensitive unique feminist methods are important to explore and understand women's embodied experiences of trauma and are essential to understand their perspectives of what drives obstetric violence during childbirth. Sensitive methods of research are crucial for the health systems to learn from and embed women's wants, to address this structural challenge with urgency, and to ensure a positive experience of care.

## Graphical abstract

Gender, power, structure and, culture can make women more vulnerable to obstetric violence. Figure 3 shows various aspects of these four cross‐cutting domains. For instance, women’s lack of choice is gender‐based and deep rooted in the cultural conditioning in and about women in the patriarchal post‐colonial societal structure, which sustains the powerlessness by keeping them in a lower position in the society.

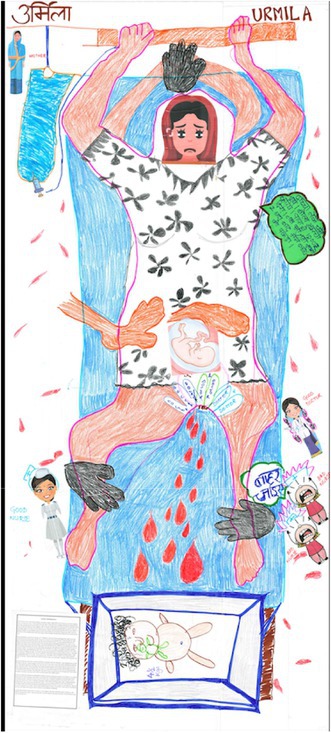

## 1 BACKGROUND

Obstetric violence is a violation of women's fundamental human rights. Yet, women might often not complain or report it. Women may even consider it an inevitable part of care during childbirth, to the extent that they might become convinced after identifying violent behavior that it was necessary to discipline the body during childbirth.

A recent literature review found several power‐related drivers for mistreatment of women, another name for the same issue, which could be interpersonal, intrapersonal, community‐related, organizational, and based on policies and laws of a country.^1^ Disrespect and abuse of women or obstetric violence needs to be understood within cultural contexts and the gender‐based discriminations, inequalities, and inequities women experience. In India, this varies by state, as shown in the National Family Health Survey (NFHS) reports, through gender‐based indicators, which show that women are disadvantaged and discriminated against compared with men.^2^ Obstetric violence during childbirth is a feminist issue embedded in the inherent gender‐based inequality that applies to the women as care receivers and midwives (and nurses) as care practitioners, who are predominantly women. It is therefore important to understand and investigate this issue from the gender lens to address the inequalities it poses.^3^

Being a woman increases a person's vulnerability to any kind of violence and victimization in India.^4^ Women are expected to function within the restrictive societal rules, customs, and norms. This is noticed in women's narratives of power and control about childbirth, and how they interact with the patriarchal structures and how society controls them.^5^ These rules and regulations determine what women should do with their bodies, and this control usually takes the power over their own bodies away from them. Any resistance is considered “misbehavior” and meted with “punishment.” Women are expected to quietly endure labor pains, screaming, or crying violates the social norms and calls for punishment through scolding and many other forms of mistreatment during childbirth to discipline their bodies.^6^, ^7^

Women's bodies and bodily purity is attached to the honor of the male, who is also the owner of her body. One way to ensure this “purity” in the hospital environment can be seen when the family ensures a particular gender for the treating doctor.^4^, ^6^ The control of the female body during childbirth in a hospital setting is a reflection of how society is conditioned to treat women at home, in the community and in general.^6^

Birth, being a natural process, has traditionally been an affair of women, dealt with at home, though guided by patriarchal norms of the society.^8^ There have been rituals around birth and pregnancy that have brought women of community together and helped them bond. The objectification of women's bodies during childbirth is stemming from the asymmetric gender powers between the women and the medical profession managing childbirth that is usually very masculine in nature regardless of the fact that often the care practitioners are women.^9^ This gender inequality is systemic and normalizes violence during childbirth to an extent where even the women being victimized start to accept it, consider it a part of the birthing process, and manage her own behavior around it, with an expectation of being abused.^9^, ^10^

Studies report that sociodemographic characteristics can make women prone to poor quality of care, on the basis of societal inequalities.^11^ This may include education level, marital status, age, gender, socioeconomic status, parity, and physical appearance.^12^ Studies carried out in India have reported poor quality of care provision to women who are illiterate or less educated, belonging to lower income groups, have many children,^13^, ^14^ are poor and come from rural areas.^15^

A study carried out in the UK found that childhood experience of sexual abuse can make women relive those experiences during childbirth.^16^ Global campaigns such as “What Women Want,” “Me Too” and “Time's Up” have opened up the platform for women to share their experiences of abuse and ask for respectful maternity care. Disrespect and abuse of women during childbirth has been called “Me Too in the Labour Room” in popular culture, with an outpouring of experiences of women from more educated sections of the society. Stories of women from rural areas and urban slums, of poor socioeconomic status and lower education, remain unheard. Understanding respect, disrespect, and abuse around childbirth from the women's perspective holistically is another gap in the literature.

All these aspects make Bihar a particularly interesting state to study women's perception of why they experience obstetric violence. In Bihar, gender‐based discrimination against women is more intense compared with many other Indian states and the Indian average.^2^, ^4^ Reproductive and maternal health is largely considered a female responsibility even when governmental policies are conceptualized and implemented. This is illustrated by the female sterilization rate being 35%, compared with 0% for male sterilization, in Bihar.^2^ Inequality in gender roles make women more vulnerable toward victimization in a society that treats its women less than men generally.^4^ Women's access to mobile phones and a bank account shows their level of autonomy, which affects their access to maternal healthcare services and maternal health outcomes.^2^ The low status of women can be noticed in a more pronounced manner from the general indicators of violence in their lives as well, it seeps into the labor room as a general culture of violence against women and the normalization of it. Intimate partner violence is common in Bihar. Evidence suggests that violence against women during pregnancy has many negative outcomes for the woman and her baby.^17^ Dhar et al. report on gender being a key factor for violence against women at home, where 43% married women are still experiencing spousal violence and 5% while pregnant. Both these indicators are higher than their respective national averages of 31% and 4%. Similar associations were found with caste and religion.^18^

There is a critical third angle to the culture of violence against women that needs to be considered, which happens in their childhood. A study carried out in 2007 found 47% of female children reported being sexually abused in India. This study was implemented in 13 states including Bihar, where the reported figure was 30%.^19^ Childbirth is a unique experience and exposes women to new procedures that may result in the reliving of abusive experiences from childhood.^20^ Research suggests that compassionate care is needed and handling women with such experiences requires the utmost sensitivity.^20^ This is yet another challenge that needs to be addressed in care around childbirth and maternal healthcare provision in general.

This study aimed to understand the drivers of obstetric violence in Bihar, from women's experience and perspectives, particularly how power, gender, culture, and structure influences the care they receive and how that is related to what shapes their perception of why they experience obstetric violence during childbirth.

## 2 METHODS

Epistemologically, this study uses critical feminist theory to inform the research question, methods, data collection, analysis, and interpretation.^21^, ^22^ In this participatory research, arts‐based qualitative methods of data collection and analysis are used to understand the deeply embodied experiences of violence in the perinatal period. Experiences of childbirth also vary based on culture and context. It helps to interpret women's experiences from the perspective of imbalance of power and position in their community in a critical perspective.^23^ There are studies that report that the issue of disrespect and abuse at childbirth can be attributed to gender‐based subordination. Since childbirth is influenced by the power imbalances in the society attributing to a person's gender, and the lived experience narrates about “women's subordination in the society,” feminist theory provides a lens to analyze women's birthing experiences.

### 2.1 Ethical consideration

The consenting process with a witness was done with the help of a consent form and a participant information sheet, which were all translated in hindi. The lead researcher begins the birth mapping exercise with a detailed description of the purpose of the study, the process of birth mapping and demonstrates the physical actions and role that the participant will take during birth mapping. When needed, the research assistant further translated the process in local dialects. Participation implies consent at each step because each participant (woman) first speaks of their experience, then lies down on the paper for a body outline as demonstrated by the researcher, and finally actively engages in conversation to illustrate key areas and ideas on the map. Given that their narratives could include sensitive aspects of birthing experience, the interviewers had access to the nearest counselor in a public hospital, in case any participant expressed the need to talk to someone about their experience. The participants were informed about this. We also obtained permission to take anonymized pictures of the body mapping exercise and to audio‐record the interview. The pictures were shown to participants and the ones they did not approve of were deleted. Seven of the eight interviews were audio‐recorded with permission.

The detailed descriptions of methods for the data collection, the challenges in implementing the birth mapping exercise, and unique approaches used for analysis have been published elsewhere.^22^ The study was approved by the Ethics Review Board of the University of Southampton to conduct this study (no. 49730).

I (lead author) am a feminist south‐asian researcher who has worked in the study setting and the state of Bihar for over a decade on multiple initiatives addressing improvement of maternal health. My positionality is important, as an Asian feminist researcher conducting research on Asian women in India, which enabled certain decisions I made in this research. My background, experiences, and context helps me understand and approach the context in which I conduct my research.^24^ Reflexive engagement is essential for this study, which involved beginning with a methods expert conducting my pre‐understanding interview before data collection, which helped to understand my fore having (familiarity and background to the study), fore sight (the influence of my background), and fore conception (what I feel I am going to find). This helped to understand the challenges on the field with data collection and find relevant solutions and structure the process of birth mapping, probe the questions when needed, and analyze the narratives and cultural context. The Bihari research assistant helped to minimize the language barrier and to ensure the narratives remain unaltered after translation and transcription to English.

### 2.2 Participant recruitment

The participants were selected from urban slums in Patna and rural villages in Muzaffarpur, Bihar. Patna, being the capital of the state, has access to specialized tertiary‐level healthcare facilities, which is more accessible for women living in urban slums. All the participants gave birth in Bihar, in public and private settings. Eight women were purposively selected to participate in a body mapping‐assisted in‐depth interview called birth mapping.^22^ A Bihari female research assistant with qualitative interviewing experience and fluency with local dialects accompanied the lead author for door to door recruitment. We requested women who have given birth in the last 5 years to participate. Participation was voluntary. Data collection challenges included hesitation to participate due to the long duration of this exercise, lack of family from husband and family members, finding space to spread the sheet and create the map, lack of time with the participants and long working hours for the researchers to accommodate participants availability.

### 2.3 Data collection

Body mapping is a relatively new method of data collection in public health research that helps to unearth sensitive embodied experiences using art.^25^ It is a fairly flexible approach. The three key components in the adapted birth mapping approach are as follows: (1) the life size body map also called birth map; (2) a one‐page summary of the participant's narrative called the birthing story; and (3) a key to understand the map called body key. All the three components are co‐created and member‐checked by the participant. Reflexive notes were written along with detailed notes about the interview environment, which was considered essential to know whether there was space and what challenges it brought in conducting body mapping with women in low‐income settings, who often lived in small houses. Between two and five interactions were done with all the eight participants recruited. Two to six hours were spent with every participant. The self‐selected pseudonyms of the eight participants are Urmila, Ria, Sujata, Pratima, Sita, Amrita, Anju, and Pairo.

We carried large sheets of thick white paper that were 6–7 feet long and 3.5 feet wide. We also carried colored sketch pens, markers, crayons, and cut outs of facial expressions, small miniature cutouts of people, children, fetus, and care practitioners, medical equipment cut outs such as injections, IV fluids, weighing machine, and blood transfusion.

A semi‐structured guide was used that included questions on personal characteristics such as education, occupation, age, number of children, and age at birth. This was followed by instructions on how to conduct the body mapping exercise and supporting questions that will help shape the birth map. The “ontological” questions on how the participant perceived “respect,” “disrespect,” and “abuse” from different aspects of their birthing experience were asked as the birth mapping progressed. No direct questions were asked on why women experienced obstetric violence, or for them to narrate their experience of disrespect and abuse. These perspectives were shared by women on their own as the process of birth mapping evolved over a few interactions in the course of a week, and as trust developed between researcher and participant.

### 2.4 Data analysis

Data were analyzed using Feminist Relational Discourse Analysis (FRDA). This was used in the analysis of the interview transcripts, audio recordings, birth maps, and birthing stories.^26^ FRDA‐guided analysis through a seven step process: (1) making chunks of data into themes on first reading; (2) reading and re‐reading for detailed coding; (3) identifying discourses; (4) identifying patterns; (5) filtering out the I‐voice; (6) listening to the recording for reflexivity; and (7) creating memos and annotations all through the analysis. Two more steps were added to adapt this form of analysis to include the birth maps: (1) applying themes and codes to the birth maps; (2) arranging the I‐poems next to the birth maps and birthing stories. This multilayered analysis provides enough opportunities for the participant's voice to take dominance in the analysis over the researcher's interpretation. The detailed coding process included generation of a codebook with in‐vivo, process, emotion, values, attribute, provisional, causation, simultaneous, and sub‐coding.

Voice‐centered relational analysis (VCRA) is embedded in FRDA and leads to the creation of I‐poems.^27^ This involves filtering out the sentences where the participant refers to themself as “I” in the narrative. The literature on voice relational discourse analysis were also referred to for creation of I‐poems. The reflexive notes and the interview environment notes were also analyzed to inform methods. The findings include the birthing stories, the birth maps, the I‐poems, and quotes made the results richer and more impactful. The birth maps and the birthing stories are member‐checked by the participants on the last interaction to make sure their voice is primary and they are not being misinterpreted. No changes were made to these after leaving the field.

## 3 FINDINGS

Four key themes were identified in analysis that led to obstetric violence, from the participant's perspective: (1) power; (2) gender; (3) structure, and (4) culture. The sub‐section presents how each of these drivers make women vulnerable to disrespect and abuse during childbirth.

### 3.1 “I am admitted under your care, so, I will have to do what you say”—Influence of power on care during childbirth

**FIGURE 1:**
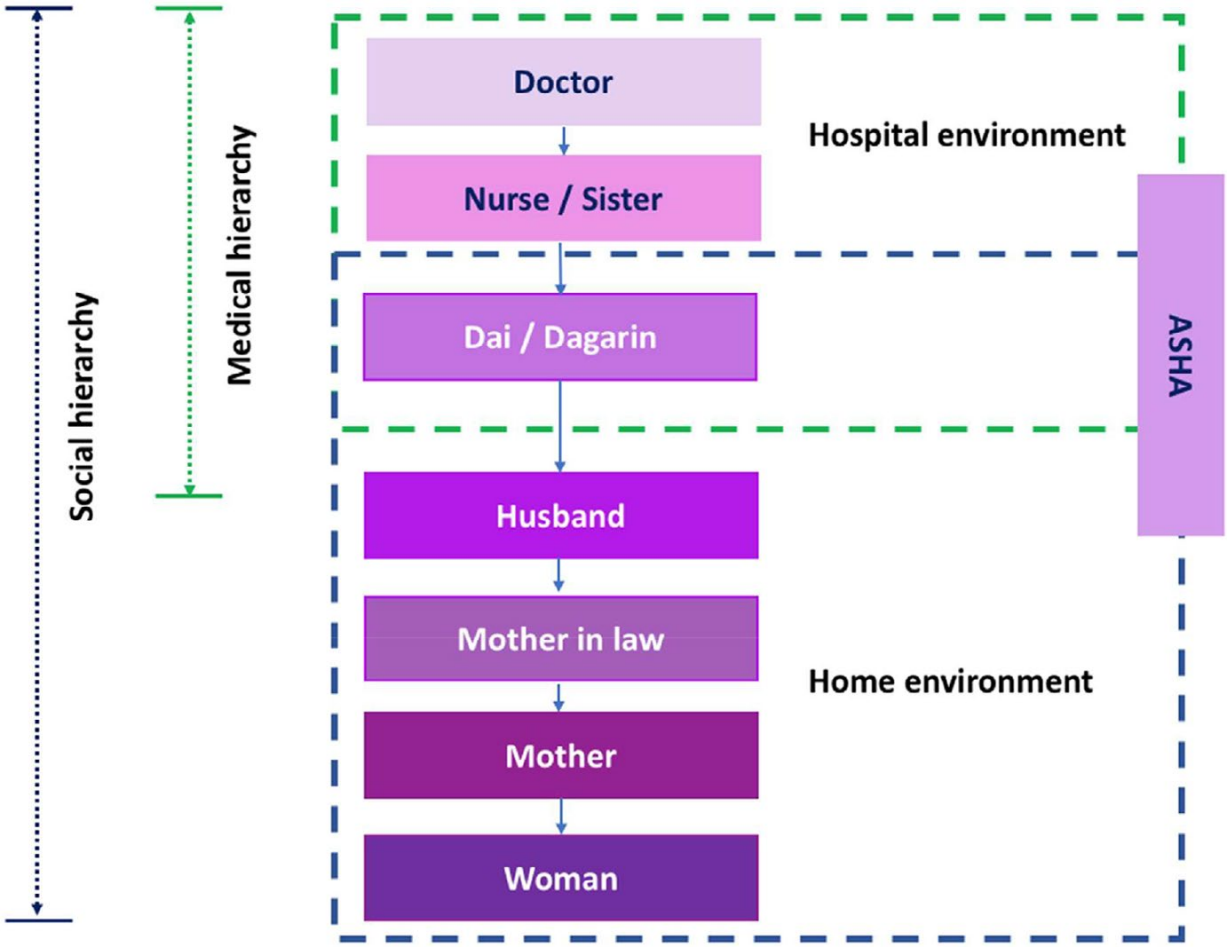
Hierarchy of power relations (authors own). [Colour figure can be viewed at wileyonlinelibrary.com]

Power was evident in the way people interacted amongst themselves in their home and hospital environment. Two kinds of hierarchies could be noted in these interactions: (1) social hierarchy and (2) medical hierarchy (which is ultimately a part of social hierarchy). Power in relationships with people increases as one goes upwards in the hierarchical ladder, and women are in the bottom of both the hierarchies, as shown in Figure 1. All the categories of people and their relations to women mentioned in the interviews are shown here. The hierarchy is created after analysis of the eight narratives about these actors at their home and hospital environments.

In the hospital environment, the doctor could be seen at the top of the hierarchy, as per the women's responses. When asked to rank all the people in the birthing room in the order of their importance, Sujata mentions that the doctor is the most important person in the birthing room and even for the care practitioners around her, such as the nurse and *dai*, the doctor is priority. Sujata is a 28‐year‐old homemaker and a mother of four, living in an urban slum in Patna. Sujata forgot to mention herself when she ranked everyone in the order of importance and power. When she was reminded of herself, she shared that she should be the most important person in the birthing room along with her baby, but agreed that that is not the reality. The inferiority can be noticed in the language women used to describe themselves as someone who will need to follow the care practitioner's orders without a choice or right to consent in the situation. This was obvious in the way women were treated like an object, and how they were often at the receiving end of the care practitioner's anger.

‘If you are so afraid, why did you conceive? What is the need of having children?’ They say such things, what can we say? I feel angry. But I am admitted under your care, so, I will have to do what you say. If I do not do as per your order, then you will again do something to harm me. So I have to listen to you…. Or they might harm me in some way. (Ria, 32 years)

They decided on their own… I was in the delivery room. We all want to have a normal birth but it depends on the doctor what they want to do. What is best! (Sujata, 28 years)

The nurse was very sad because we were creating inconveniences for them early in the morning. (Amrita, 22 years)

This was manifested in the way communication between these actors played out. Doctors and nurses did not directly communicate with the women and did not feel the need to explain the procedures to them and treated them as an object. The most extreme case was seen in Pairo's first cesarean birth, which is also shown in her birthing story (Box 1).

BOX 1 **Pairo's birthing story**

My heart was beating out of my chest, because I knew what was going to happen next. He held my hand tightly, a stranger, but it felt good. As if someone my own is keeping me calm. Scared, I asked him to press his hand on my chest, on my heart. I am a 29 years old, government school teacher and this was my second childbirth a year ago.

Memories of my 1st birth had traumatized me. Everything is still fresh in my mind. Even now when I think about it, I just know, never again. I wasn't in pain but I was leaking some fluid. So everyone took me to the government hospital that morning. There were many women all waiting for their turn, and then I saw that doctor wearing a plastic glove checking everyone in that dirty environment. I ran from there! I was taken to a private hospital next. The lady doctor just made the nurse lift my petticoat and nightie up; and forced her hand inside me without any explanation. I started screaming and crying out of pain. “You can never have a normal birth, if you can not bear this pain.” The next 3 days I was in observation when I was given 19 bottles of fluids, many injections to increase the labour pain and numerous vaginal examinations. The nurses would just come and insert their hand, not even minding the crowd and how many people are around me. I was frustrated and complained to the doctor, “why does everyone has to first insert a hand inside me, without even talking to me. Is there no other way to check?”. She said nothing. My mother says, “women have to endure that, to have a child”. Even now sometimes I tell mummy, “that wasn't right!”

I was in the cafeteria with my family when the nurse came and just dragged me by my hand to the operation theatre. No explanation given! My family stayed outside the OT. There were 8 men in the room all in regular clothes, like they are on a picnic! One of them said, “get up!”. Gave me an injection on my back and made me lie down. No explanation given again! That's when I realized I am going to get operated, no one told me. My only solace was that there won't be any labour pain. Another man blindfolded me because the less I see, the less uncomfortable I will be. I felt someone talking my petticoat off and lifting my nightie to my chest. They were treating me like a doll… or like an animal… doing whatever they want… not caring about me at all. Like I did not exist! I was filthy and my hair tangled without a shower in 4 days my clothes getting drenched in my fluid and drying on me. I did not know anyone in that room. I asked about my lady doctor to this other guy who was apparently her son. She arrived later.

They played music. It was calming. There were other sounds too, of instruments and scissors cutting through me like they are cutting a jute rag. Everyone was talking amongst themselves while they took the girl out of my body. It's a girl, they discussed and I thought, “I will tie her hair in two pig‐tails and take her to school with me.” I stayed in hospital for 10 days after that because I had fever and chills and was recovering from surgery. Meanwhile the baby's doctor did not tie my baby's cord properly which kept bleeding. She got infection the same night and my husband had to take her to another hospital, 3 kilometers away, every day for injections. I struggled to breastfeed my baby and even hold her properly.

I cried when I could not have a normal birth the 2nd time with my son, 2 years later. The doctor pressed on the incision and it hurt. “it can get torn and you might get a cut down there anyways. You'll need a big operation.” She said. The normal birth's pain lasts 4 days but the misery of CS lasts for years and breaks your body. In the beginning sometimes the incision used to hurt like someone rubbed *chilli* powder on it for the medicine I was prescribed to apply on it to get rid of pain and redness in the first place. This was a quack in our village who considers himself our area's MBBS!

My husband asks, “why did you not get sterilized if you don't want another child?”. “You get sterilized,” I tell him. He makes excuses that he'll get weak. So we both don't get it done. But I do tease him saying, “my life is in your hands” when we get intimate. I feel I needed to share these with someone, it all needed to come out as I could not talk about it with anyone. That day somehow got over, but those haunting memories have stayed with me.

Pairo felt that the doctor was overconfident and did not care about what her patients might be thinking of her as she prescribed medicines without describing what they are for and just expected her prescription to be followed without any question. She also felt that another doctor she came in contact with for her newborn made a mistake that she had no right to feel angry or concerned about because he was treating her baby, and due to that he had some power over her. This is the same pediatrician who scolded her husband after having been at fault himself. A display of physical overpowering is noticed in restraining women during various interventions where people around birth from home and hospital participated in holding the birthing woman down. Women did not like this, but considered it normal and did not complain about it. Power was displayed by completely ignoring the adult birthing woman's presence in the room and not giving an option to voice her experience by cutting all communications with her. The communication instead took place with “surrogate decision makers”^28^ who converse on the behalf of the birthing woman and make all her decisions where the woman has not necessarily appointed anyone to do it.

They were talking about me, around me, but not to me. They were only talking to my parents. They were informing them after checking me. I did not ask them anything. What is the point of me asking them things unnecessarily, the elders are there for that. They need to know and decide things. (Urmila, 25 years)

She did not need to ask my name. She was calling me ‘you, you’. (Sujata, 28 years)

I wanted to straighten my leg for a while but everyone was holding me down, my legs and hands from all four sides tightly. I could not bear that, the touch of anyone. (Urmila, 25 years)

At home, women followed the decisions of their *guardians* without questioning. Father at the *mayka (*maternal home) and husband in *sasuraal* (in‐laws place) are the people with the highest authority and power. In the birthing environment, the father and husband have a limited role but the oldest women such as the mother‐in‐law and the mother often make the decisions about pregnancy and childbirth. They negotiate that women should not fuss about the interventions and encourage endurance. This is seen in some of the quotes in gender, culture, and structure as well. *Dais* and Accredited Social Health Activist (ASHAs) have a dual role to play, evident from the ASHA's placement in bridging the gap between home and hospital. *Dai* was often seen providing care in both these environments.

She (ASHA) told me ‘you might have withdrawn the money from your account.’ … I gave her my passbook and told her to check if she does not believe me. (Amrita, 22 years)

Participants often did not question the authority of anyone and followed all the instructions without resistance. They went back to the same care practitioner who abused them, without confronting them. There were no consequences to the abuse of power people in the home or hospital environment exercise over women.

He was a good doctor and we knew that he made a mistake… he scolded my husband… I just feel he did not do it intentionally. I trust him so much that I still go there for my child. But I never told him. Did not want to hurt his feelings. (Pairo, 29 years)

They are doing their work, they are not at fault. They were doing as they should do. According to my level of pain, I was also behaving a certain way. At the time of birth, they used to remove the cloth and lift it up but that is necessary. (Sujata, 28 years)

The fact that the “surrogate decision makers” did not feel the need to convey to the birthing women any details about the communication they had with the care practitioners about the course of treatment or decisions and choices made about the woman is astounding.

I was thinking these people would think about my wellbeing only. They'll have my best interest in heart, my mother and husband. I believe they should have told me about it but they did not. What should I say, I was just a patient. But these things should have been explained to me. (Pairo, 29 years)

Much of the exercising power over can be seen in Sujata's birth map in the number of hands that have conducted vaginal examination and uterine exploration by *dais* who are not trained for such interventions, the extortion and demands of “happiness money” by trained health care practitioners and the lack of a chosen birth companion.

### 3.2 “I was blindfolded … because there were men”—Influence of gender on care during childbirth

Gender manifests in many ways and is a key factor that resulted in the imbalance of power between different actors shown in Figure 1. Son preference is expressed at multiple occasions by the people from home and hospital in the birthing environment. This is part of the *Bihari* culture, as in many other parts of India beginning at birth and influencing every part of the participant's life including their education, financial independence, occupation, marriage, and every role they play. Care practitioners demand more money when a son is born and offer a discount on the birth of a girl to make up for the family's sorrow.

Nobody was playing with my girl. No one was taking her in their lap (when she was born). Then slowly people started warming up to her… my mother‐in‐law was very happy that it was a boy. (Amrita, 22 years)

If it is a girl baby they ask for less money, it's more for a boy. If someone gives birth to 2‐3 girls then they don't ask for bakshish (happiness money) because it's an atmosphere of sorrow for the family. They (care providers) understand the situation when to ask (for money) and when not to. (Sujata, 28 years)

Women often have no say in the major decisions of their life, regardless of their level of education. This was reflected in their decisions about pregnancy, birth, and family planning in general. Implementing family planning in the home environment was treated as women's business, and women accepted it as they accepted domestic work as women's domain. This meant it was naturally the woman's body to be sterilized if a permanent method of contraception was decided upon, and this decision was made by everyone in the family including her husband, but not her, as can be seen from Pratima's narrative. Women did not participate in money‐related matters, they had no say in expenses and often did not even have a bank account. The money they get from the Janani Suraksha Yojana (JSY), a scheme that incentivizes women from institutional delivery, was spent on matters of the house.

My work is to just cook and take care of the house. So I don't know much. (Sita, 22 years)

I feel two kids are enough, but my husband want's one more. As in, a son!… no, no, no… how can a man get sterilized?! (Pratima, 19 years)

Women are not immune to these gender roles, and it shaped their perception in terms of occupations as well where they often assumed a woman in the birthing area is a nurse. They also assumed men to be nurses in OT to ensure oneself that their presence there was necessary.

What will I call them, gents nurse? Are they nurse? I mean compounder may be? (Pairo, 29 years)

The doctor played music on mobile and was changing clothes so I asked where is madam? He said ‘mummy is coming!’ The doctor whom I used to show was a lady but her son came in the operation room… he had a degree… I did not know he is a doctor. (Pairo, 29 years)

Women cited many reasons for the disrespect and abuse against them during labor and birth. They felt respect was for the rich and if one has the financial condition to pay the “happiness money,” apart from bearing the cost of care, their “patient” will be cared for well. A “guardian” accompanying them showed there are people who will pay. An experience of previous birth mattered as well, because women would have experienced labor pain and often obstetric violence before, so would behave accordingly. Poverty and a lack of education invited disrespect, according to participants.

When poor come for care, they get scolded. We are poor, uneducated, weak and its our need that we went (to the hospital), so we can not say anything and have to listen to everything they say. An educated person knows how to talk to them… an educated person will be able to reason well with them but we can't. We have to bear the brunt of their anger. (Amrita, 22 years)

The one who screams gets abused! They are not using their strength to push but to scream. They'll have to listen to abusive language. Those who have a habit of birthing they do not have to listen to such things, but new mothers don't have a habit of birthing, so they don't know much, they have to listen to such things. So, they were abusing me. (Ria, 32 years)

I was blindfolded because I would have felt uncomfortable, because there were men. There were no ladies. The doctor who was going to perform the operation was also a man. (Pairo, 29 years)

When women asked for an explanation or questioned a procedure or medication or the duration of labor, it often fell on deaf ears. This passive‐aggressive treatment of women in the matters of their own birth is gender‐based, and women understand that they have no right and say in their own matters. Women's pain is not addressed or acknowledged in most scenarios.

I kept asking for anesthesia but they were not even listening to me and kept doing it (episiotomy repair)… I kept screaming and everyone was holding and stitching me. I didn't have as much pain in delivery as I did when they stitched me. (Urmila, 25 years)

I do feel that the bad experiences were with my body. So, those things don't matter to me anymore. What happened, happened with my body but my spirit is untouched. (Pairo, 29 years)

**FIGURE 2:**
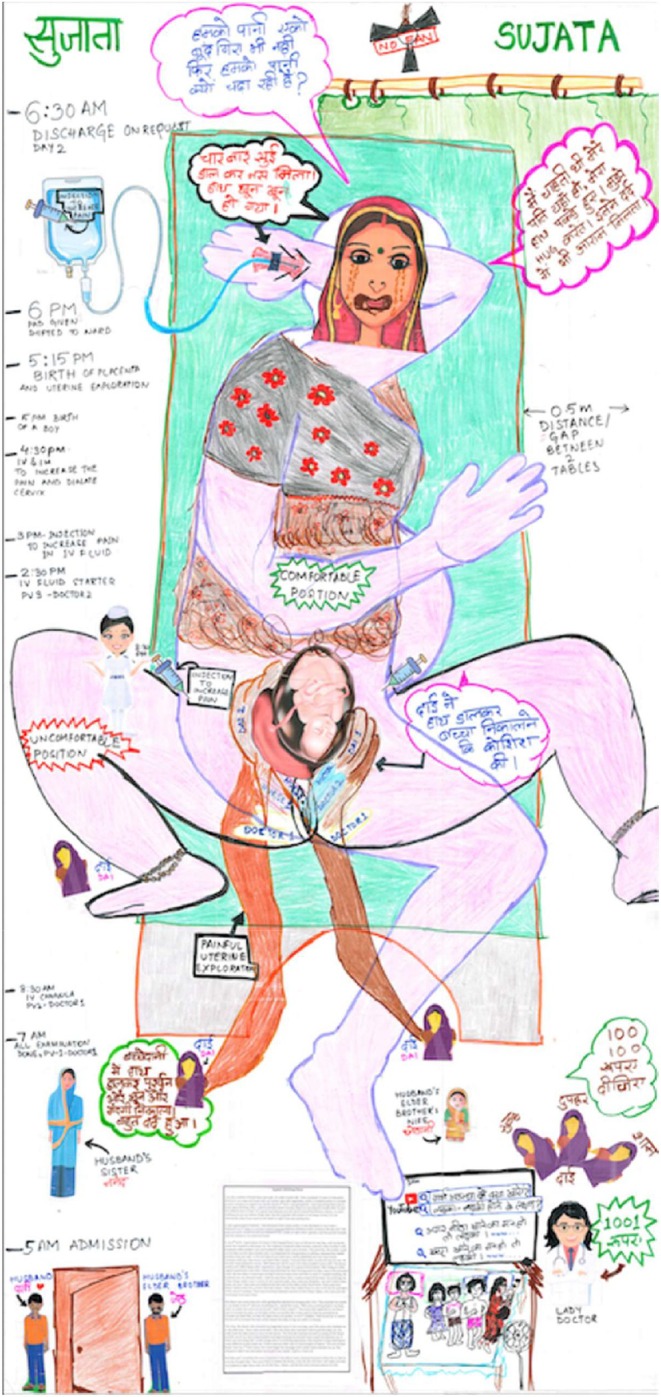
Sujata's birth map. [Colour figure can be viewed at wileyonlinelibrary.com]

The manifestation of gender in different aspects of a woman's life can be seen in the I‐poem (Box 2) from Amrita's narrative titled “I.” Amrita's birth map in Figure 2 represents some of what is shows in her poem as well.

BOX 2 **Amrita's I poem—“I.”**

I

I might have been 18 or 19 years old. I have a husband.

I run my shop, take care of my children and my house.

I go to nearby PHC. I go there for medicines and all. For every single thing, I go there. When my periods stopped, I went there to get checked. Even for delivery, I had my boy there.

…I fall sick, my child falls sick. I am fine now. I have taken medicine at night.

…my body aches a lot, sometimes I get headaches at night, I get fever. I don't know whether it is due to tiredness or something else.

I was very healthy before. I had no fever, nothing. I take medicine and stay well for a week and then again I fall sick.

You can see how much garbage there is in my home due to falling ill? I am all alone. If I fall ill, there is no one to look after me.

My mother in law used to stay here. No one is here to look after my kids and family, I only look after them.

I go to the market for purchasing the things for my grocery shop. I sell it. I leave my children at the nearby centre. After cleaning and dressing them up, I leave my children with them. I get help. I leave the children with the women in my neighbourhood. They help me a lot. They treat me like their own sister. Doesn't matter, whether they are elder to me or younger.

I was alone that's why I used to do all the household work along with taking care of grocery shop. It used to hurt a lot when I carried jute bags of rice, which is usually very heavy.

I use to lift 25 kgs of jute rag. I use to sit at the grocery shop. Everything was done by me.

I was almost 8 months pregnant.

I used to call my sister for household work such as cooking and all. I took rest then.

I had a 2‐year‐old daughter, whom I use to take care of like washing clothes, cleaning her, bathing her. I use to keep her with me.

I used to get a lot of rest. Seriously, when my sister was here, I used to get great help. She did all the work after getting up in the morning like cleaning, sweeping, washing clothes, cooking and used to keep me and my child healthy. Used to bring meal and medicines near to me and I just had to eat. My sister used to do everything.

I took 3 days to give birth to my first child, since I was older (18 years) when giving birth.

I used to be very sick with the second child in my womb. I did not like anyone talking to me. I used to always feel tired.

I used to be lethargic. I could not even eat much food or water. I didn't like anything. My mouth felt dry but at least my sister took care of me nicely, so I relaxed. I also had nausea and vomiting during my antenatal period.

I mean my lips and tongue became red and blistered and mouth was dry.

It was my wish to go in a saree. I wore a saree for both of my births.

Initially they (care providers) were very angry but when they saw that I had baby boy, they all were so happy and they started talking with me politely.

In my first childbirth, they were all from my mother's house and everyone was very happy.

They were very happy because when I had the girl child, everyone was happy at my mothers' side. Few of my relatives were working in hospital, so I didn't have much trouble in my first baby.

My mother in law is like mother only, right? She was like my mother.

During my girl, only my mother in law was there from in laws side. She was not happy that I birthed a girl.

No body was playing with my girl. I used to do everything for her, put a kaala‐tika on her forehead and put her to sleep.

At the time of my boy, the nurse was there. The nurse and dai were angry, but my mother in law was happy for a boy. I was also happy. I was happy both times.

I used to get a lot of pain while passing urine.

I might be dark skinned but I am very neat and clean.

I tell everyone how I gave birth. It was God's grace that I had normal birth. Whatever I had, it's good. I told them what problem I had. Sister should come and check me completely and tell me about my condition, how much time it will take for me deliver.

I will feel respectful when they will do my delivery on time without much delay, when they will speak to me politely with a smile. When they will take care of me nicely. They should treat me like family member. No matter whether I am birthing a boy or girl, I should be treated well. I want sisters (nurses) there to check me nicely.

They were not calling me by name but they were calling me like “babu, beta, everything will be fine”. They were scolding me a lot, but were talking to me nicely. If they talk nicely with me then only others will come there.

### 3.3 “The more money we give the more convenience we get”—Influence of structure on care during childbirth

Healthcare systems about childbirth follow a societal structure that includes certain norms and cultures, and they are deep‐rooted, and influenced by gender and power. Women, couples, families, and communities are conditioned to these structures that could relate to their home, extend to the birthing environment and go beyond. This was noticed in their narratives where they wanted to follow something because it has been followed enough times around them, for them to normalize it. The structure of birthing may include many good and bad aspects that condition choices or the lack of it in maternal health in general and specifically during labor and birth. This structure encourages further deep‐setting of the discriminatory culture of doctor‐knows‐best which makes women and their families follow care practitioners without question or communication.

We all think we should have normal birth. That's the best! But after going to the doctor, it depends on her what she will say. (Sujata, 28 years)

My mother‐in‐law said if she doesn't check you down there how will the baby come out?’ we don't understand this but they do. (Sita, 22 years)

Sujata feels what the doctor and other care practitioners do in a birthing environment is in the best interest of the birthing woman. It might not be comfortable, but women must go through it because they do not have a choice to deny care. Participants believed that care practitioners know what is best for them, but hoped that their respect, dignity and comfort would be considered when making choices for them and that they should be given a choice. For instance, Urmila believes that babies are exchanged at government hospitals based on sex. Women have assumptions about the quality of care they should expect at government and private sectors and also at different levels of care provision. This guides their decisions and plans around birthing, starting with wearing a petticoat to seek a little privacy in a structure that provides none, to choosing where they want to give birth.

In government hospitals they do not cut open the belly unnecessarily. (Amrita, 22 years)

People say about government hospitals that you get the facility that you can afford to pay for. The more money we give the more convenience we get. If you go to private, they will rob you. You have to bring everything yourself in government, but you don't need to pay the doctor. (Ria, 32 years)

Those who are accompanied with their guardian are treated well… if they feel that they wont make money after birth from guardians, then they do not talk well… otherwise they do not give any attention. (Sujata, 28 years)

Calling an ambulance and ASHA when in labor is part of the structure and so is expecting the JSY incentive after birthing in a government hospital.

We used it (JSY incentive) up to buy knick knacks for home. For vegetables and other important things. If you have money in your hands, so many things will get over with that. Slowly we used up the money with multiple things. (Sita, 22 years)

Disrespect and abuse of women is structural and normalized. Women expect some extent of disrespect, abuse, or the absence of respectful care. They accept it at a certain level as well, but do not object to what is unacceptable, as complaining is not desirable. They come to receive care knowing many aspects of birthing practices that they do not necessarily like, but the consensus is that they have to endure it to give birth, every woman goes through it. They share many arguments that support this belief which includes their lower status, low level of education in comparison to the care practitioners and the fact that they are care seekers, so the care practitioner has more power, which is a part of the structure.

He said get up and injected in my waist taking my clothes off… they were treating me like a doll. They were not taking my permission from me and did not have to say anything. No explanation! (Pairo, 29 years)

I quickly drank it (soda) as I had ordered it, but those people (hospital staff) wanted it too. It happens in government hospital that people ask you to bring something to feed them. (Sujata, 28 years)

I left my education when I was 13. But it's their (parents) responsibility to send me for further studies which they did not do. They should have been stricter. (Urmila, 25 years)

**FIGURE 3:**
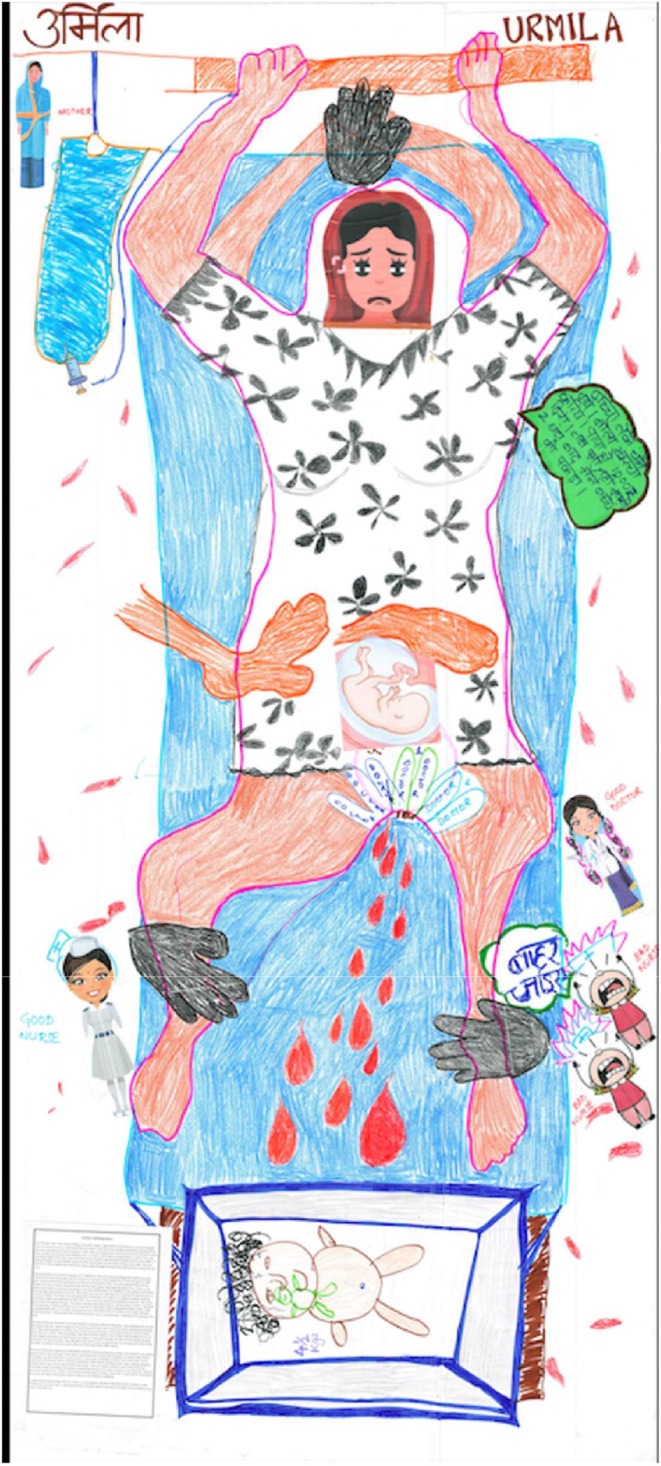
Urmila's birth map. [Colour figure can be viewed at wileyonlinelibrary.com]

The structure of society shapes people's attitudes and behavior about how women should be treated in general. Their education, mobility, financial independence, and decision‐making rights and choices are all part of the societal structure. This has a heavy influence on how women are treated in the hospital and all the actors shown in Figure 1 are part of the same structure. Urmila's birth map (Figure 3) and birthing story (Box 3) presents this well about her life.

BOX 3 **Urmila's birthing story.**

I am 25 years' old. I have three children, all under 5 years. I gave birth to all of them in a private hospital near my maternal house. I had heard from my sister in law about her horrible experience of giving birth in the most reputed government hospital in Bihar. She was always alone and had to shout for attention every time, as they were always busy chatting. Also, no matter which baby you gave birth to, a girl or boy, you can bring a baby of any sex, if you have the money. People in government hospital wait for your death. In private hospital we pay 20,000 rupees for birth, but at least we can ask for things as our right, since we paid for it. All my babies were born from down under through *tiny operation* (episiotomy), except the last one.

My grandmother had told me during my 1st childbirth, that you'll leak something from where you pass urine and the first three pains will be wrong. You must go to the hospital the 4th time you get the right pain. My second birthing experience was the worst among the three. The same people delivered my babies, the same doctor, one good nurse and two bad nurses. They kept shouting at me, where as the other nurse calmly instructed everything. They kept talking about my childbirth to my parents and others and amongst themselves, not to me. The doctor was nice too. She kept me covered and exposed only as much as needed. The doctor did everything from checking in my vagina if my baby is coming, to giving injections, fluids, and stitching me after birth. The nurses just cleaned everything around me. The nurses were there throughout though. The doctor had just instructed them to ‘call me when it's time’. I was given two injections, a tablet and there was an injection in the bottle which increased my labour pains. I kept screaming after that, as my pains increased so much. My father told my mother to be inside, because I might be scared. I did not want her to touch me since I was in so much pain. I wanted to hit everyone. Her too!

My third birth was the best! It was the shortest in duration with very less pain. I was waiting to be cut, but the doctor did not. I was so happy! In the previous birth they said it wont hurt while cutting and stitching me. They said it was just 2–3 stiches, but I know it was much more. I knew when she cut me *down there* and I felt every stitch. I felt everything. They held me down tightly! Two of them held my legs and one of them tightly held both my hands above my head as I screamed through the pain and the doctor ignored my pleas and kept stitching. They said don't you want to give us something out of happiness*?* They finally brought my baby to me hours later, after my father gave them 2000 rupees.

Have you done any research on alcoholism? Alcohol ruined my family you know! I am living for my children and I want to start earning to raise them to be away from my alcoholic husband. He drives a taxi, drinks and beats me, but I took him to court and he is now in jail. I probably shouldn't have done that. He has money for alcohol but not for children's education. I don't know anything to get a job, I stopped going to school when I was 13 and was married very young. I was very happy without a care in the world, but marriage ruined everything. My parent's should have forced me to stay in school, it was their job. I have an account in bank but no money. I don't know anything about money, I never did. I never ask for anything for me, but I can fight with anyone when it comes to my children's wellbeing.

I never shared these things with my mother or my husband. We don't talk about birth in family or with friends, you just don't do it. I did not know that I had the need to talk about my birth. You are closer to me than my family now.

### 3.4 “How could I ask him, how it will come out?”—Influence of culture on care during childbirth

**FIGURE 4:**
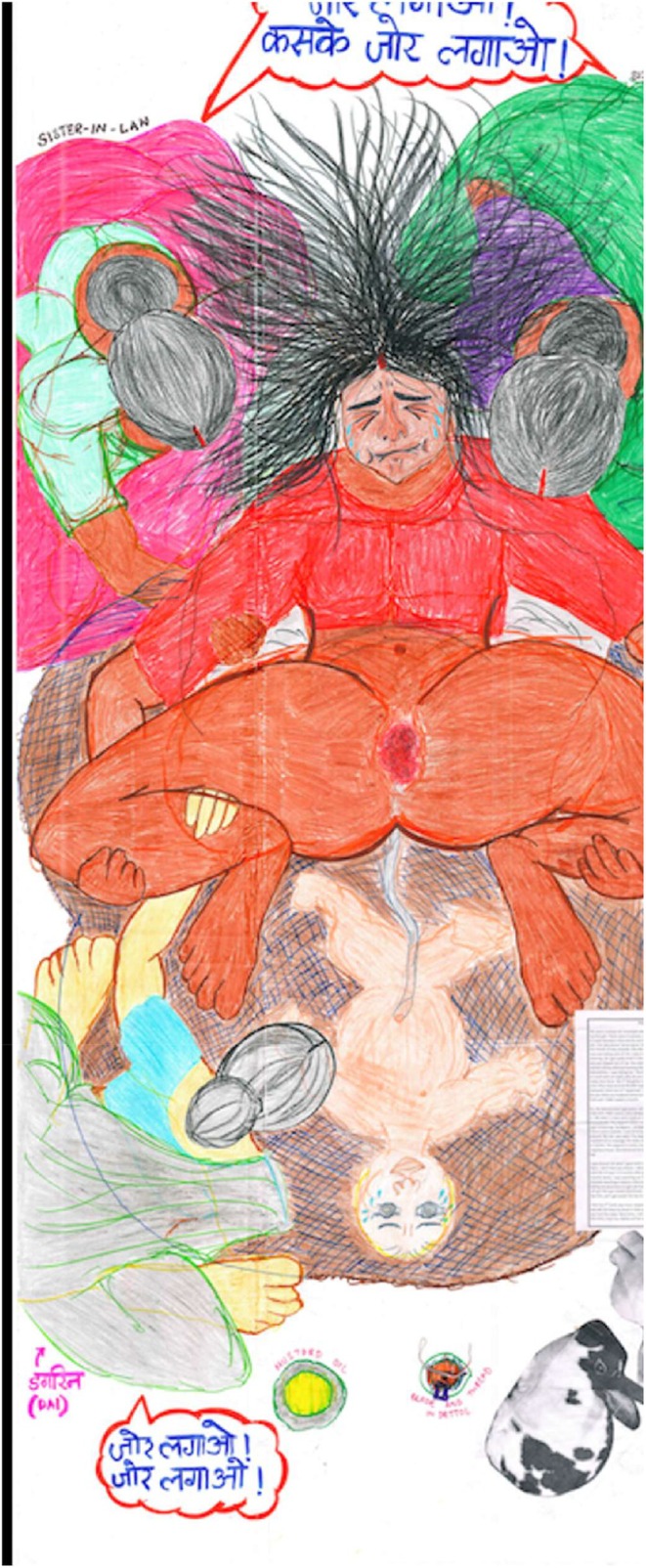
Pratima's birth map. [Colour figure can be viewed at wileyonlinelibrary.com]

Culture guided many actions around childbirth. A family could have certain cultural norms, myths, and traditions that could be limited to the family or extend to the community or the state or country, based on the elements that bind them together which could be family ties, gender, caste, religion, and so on. Culture also guided the people accompanying the woman giving birth. The presence of a *dai* is part of the culture. They are noticed in all the home births in rural or urban participant's interviews, even in the hospital births, although there is no role of a *dai* in the hospital birthing environment and yet she seemed to be one of the key care practitioners. The dai's presence and role can be observed in Pratima's birth map (Figure 4) in a home setting. How and where one gives birth also becomes a part of the culture and is thus aspired.

Everyone wanted it to be normal because nobody had given birth by cesarean in my family before. Nobody wanted me to have a cesarean. That's why everyone was sad, my mother was sad and crying and saying ‘what had happened to my darling girl!’. (Pairo, 29 years)

Culture drives many small and big actions around childbirth. Women's culture dictates the wearing of sarees in general and when they give birth. Wearing a saree and/or petticoat under it, helps to avoid overexposing them when giving birth, be it at home or hospital. Many care practitioners recommend women to wear a nightie when they go into labor, for the ease of wearing, removing, covering and uncovering the legs when needed. Not all the customs and cultures were healthy, some were quite obstructing for the community and had drastic influence. Son preference is a dominant culture in Bihar. This often turned the birth of a girl into an atmosphere of sorrow. Even though sex determination in pregnancy is illegal in India, people often try to determine sex of the baby. On denial they may feel that they are not being told because it is not the preferred sex.

For 9 months I assumed that I had a daughter because she (doctor) won't reveal it to me. So I thought it's a girl because she wasn't telling me…The doctor said that this is a girl. People want their first child to be a boy. They also say if it starts walking late it will be a boy. (Pairo, 29 years)

I thought I will have two boys and a girl and then I will get operated. But this is also a girl. I usually stay unwell so I don't want any more children. (Sita, 22 years)

It is essential to understand how the culture of abuse penetrates into people's lives when they are growing up in their surroundings, including the home atmosphere. This aids in normalizing violence against women, where they adapt to it as part of their culture and thus do not resist violence during labor and childbirth. Extortion is a kind of abuse during childbirth, has become a part of the culture around childbirth in Bihar.

Some people give it happily and others have made a tradition out of it. Like those people who don't want to give or can't afford, they ask from them as well. Sometimes they ask again and again to people who readily give them money once. Now that's greed! (Pairo, 29 years)

Silencing women, treating them unequally and their overall lack of importance, is cultural. This silencing is commonly seen in women's endurance for pain. Women are supposed to endure pain and they do that as long as they can. Their silence about anything that happens in the birthing environment is part of this culture as well. Women understand that they are not supposed to have any conversation in the birthing environment.

I was feeling bad but I could not say anything because we were in the hospital. If I say anything to them then they will say ‘you are not the only one having a baby here. There are other people too who don't have any problem. You don't have anything special down there’. (Ria, 32 years)

Everything was happening in front of me but I could not say anything. You are not supposed to talk about operation … you lose a lot of blood and the body becomes weak. (Pairo, 29 years)

I first tried to bear the pain but when it was unbearable for me then I told my mother. (Sita, 22 years)

Birthing is considered women's business, but ironically, the woman giving birth has no say in it. The women accompanying the birthing woman manage most things. Women often go to their *maayka* (mother's house) to give birth mainly expecting some rest during the late stage of pregnancy and immediately after birth.

We don't do any heavy work, we just cook food after birth. No lifting heavy things. (Sita, 22 years)

I was at my mother's house… there was no one to take care of me here so I went home. There my mother and sister‐in‐law to take care of me. That's why I went home. (Sita, 22 years)

Women do not talk about birth regardless of their curiosity. They are surrounded by women who have given birth but do not share the gory details of the birthing process and women accept it without question. They do not discuss it with their husbands owing to shame. The culture is to not talk about birthing and births in Bihar, it is stigmatized due to its connection to exercising one's sexuality.

At the time of birth everybody keeps searching for all this (information). Everyone is curious to know all this. (Sujata, 28 years)

How could I ask him (Husband) ‘how will it come out?’ (Pairo, 29 years)

What will happen is God's will. When God will want it to happen, it will happen. (Pratima, 19 years)

The lack of importance to women, that begins at birth, is cultivated through living a life that is not valuable. A date of birth seems of less importance which was not celebrated. Decisions are made for them and they are supposed to follow. The I‐poem from Ria (Box 4) looks into this aspect of their upbringing which also mentions how important it is to be a girl of light complexion to be considered marriageable and to give birth to a son thereafter, as soon as possible.

How old am I? I don't know. (Sita, 22 years)

I never took any decision for myself neither for my childbirth.” (Urmila, 25 years)

BOX 4 **Ria's I‐poem “If I had been fair and had birthed a boy.”^28^**

‘If I had been fair and had birthed a boy’.

I would have been happier if I had a son. I have no son or brother at my home. I had one sister, she passed away at the age of 3 ½ years.

I have never seen my father. When I was 1 ½ years old, my father passed away and I was all alone at my home. From then on my mother took care of me as I grew up.

He was murdered. That's why I thought if I had a son then it would have carried out my family name for another generation.

I was expecting to have a son but then I had a daughter. I was sad. I swear I was sad. I still wonder why I did not have a son. Why did I have a daughter.

I live in this house and no one asks me where I work. They put allegations that she might have done this or that. People gossip about ten different things and spread rumours behind my back. Everyone says that she has kept him, him or him. What to do? I feel like crying. Whenever I go to work, people say that I am going to do wrong things.

That is why I thought, if I had a son, then he would have been my support system. I should have had a son.

Everyone was happy that I had a daughter.

Then I have also said that one has done it by begging, another will also do the same way. I was married with great difficulty. I used to tell mother, ‘I don't want to marry him.’

My family ruined my marriage. My uncle said that the girl works in a boutique and she works in a parlour. I used to be very late. It started from 11 a.m. to 8 p.m. Sometimes it was 9 p.m. in the evening. That's why I don't work there.

My husband started doubting me. I said I will not stay with him.

If I was fair then I would be beautiful, my mother would marry me somewhere nice. I would have been educated, I would have been better off. My luck was bad!

God could have made me fair and send me with all the qualities.

## 4 DISCUSSION

**FIGURE 5:**
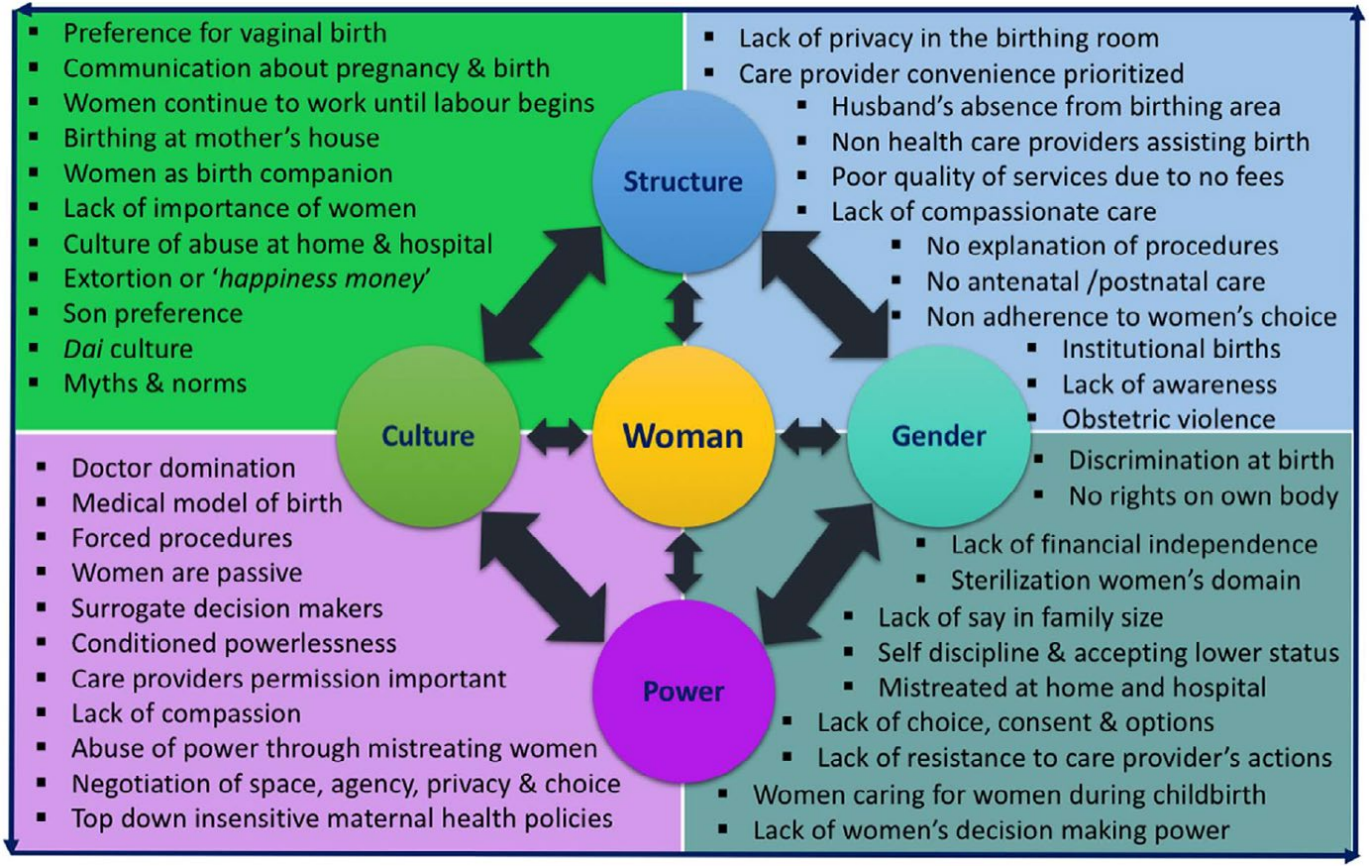
Influence of gender, power, culture, and structure on labor and birth (Authors own). [Colour figure can be viewed at wileyonlinelibrary.com]

Gender, power, structure, and culture can make women more vulnerable to obstetric violence. Figure 5 shows various aspects of these four cross‐cutting domains. For instance, women's lack of choice is gender‐based and deep rooted in the cultural conditioning in and about women in the patriarchal post‐colonial societal structure, which sustains the powerlessness by keeping them in a lower position in the society. Similarly, the other aspects have been put in one domain each, based on the researcher's understanding of which domain each of these aspects represents best.

Women are often reported as powerless in terms of themselves and their body, including during pregnancy and childbirth.^14^, ^29^ This begins with the uncertainty of whether she will be allowed to be born.^30^ Throughout the life course, women are not allowed to make decisions about their education, financial independence, age of marriage, or partner, and they have no say in conception or in the methods of contraception, about family size, about giving birth, where to give birth, or whom to have as a birth companion. Also, crucially women do not have a say in the interventions, which include many procedures that put them through obstetric violence that they are supposed to endure silently. This includes being conditioned to quietly endure labor pains and not getting a response to questions and being uninformed, even when being operated or not given a say in the care of their newborn. This silence around childbirth and lack of decision making power is in line with the literature from India and other countries. Their silence and resilience to endure violence in the hospital environment in accordance with their intimate partner violence and domestic violence in their home environment in Bihar.^4^, ^31^

There are examples in this study that broke this narrative of powerlessness. Sita refusing to let her *Dagarin* (traditional midwife) mother‐in‐law do a vaginal examination and deciding to give birth at the hospital, in an area where most of the births in the neighborhood were traditionally assisted by her mother‐in‐law, is one such example of exercising choice. In another scenario, Urmila wanted to hit her mother when she was her birth companion, this goes against the culture and structure of birthing in Bihar. In their personal lives, both Ria and Urmila share about intimate partner violence, and both have taken a stand against it, Urmila by sending her alcoholic husband to jail, and Ria by divorcing her husband without an alimony. These are rare scenarios from India, where the rate of divorce is 0.24% of the married population.^32^

Receiving respectful care, through respectful communication, changes that power dynamics and equalizes the imbalance where women feel they are being treated with kindness and compassion, which leads to a trusting relationship between the care practitioner and care seeker.^33^ The findings suggest that women are persistently negotiating power at home and hospital. At the hospital, she is aware that she is at the bottom of the hierarchy by structure, so she overlooks the disrespect and abuse, and does what is right for her baby, after everyone's choices aligning with the conditioning of going home with a live baby being the ultimate desired outcome. This power dynamic is more equal, if looked at equality as a spectrum, in home births.^34^ Education or financial independence did not influence this power dynamic much for Pairo, who wanted to be told about the interventions and also deny vaginal examinations, or at least request for privacy. Although, she could not state any of these to her family members or the care practitioners. The influence of her education and financial independence could be the reason for her decision to not give birth in a government hospital and seek care at a private hospital.

The culture of silence is seen in most aspects of women's experiences which they accept and endure in their usual lives that reflects in the birthing environment as well.^35^ The norms around birthing is that women are supposed to endure all of it as the usual course of institutional birth, and does not fall in the category of violence or victimization to be grieved.^35^

Obstetric violence is gender‐based violence. The intersectionality of a woman's other background characteristics such as education, socio‐economic status, gender, marriage, religion, age, gravida, caste, class and the difference between her characteristics from that of her care practitioners, may make her more vulnerable to disrespect and abuse during childbirth.^6^, ^14^, ^36^ This has been reported elsewhere, as scheduled caste women were seen to receive treatment after higher caste women in Bihar.^36^ Literature also suggests that access to medical education has traditionally been limited to mostly men of higher caste and richer families which increases the social distance between the woman as care seeker and her doctor.^37^ In this study, women were abused due to some of these factors as well, which were also identified by the participants such as poverty, lack of education, being a woman and their marital status.

Structural intersectionality explains why the women are at a disadvantage in a patriarchal culture, in a male‐led medical model of care and through the various factors that maintain the dominant power of men over women. The current structure of birthing has shifted from home to institutional births as a dominant culture even in lower socio‐economic settings with the implementation of Janani Suraksha Yojana (JSY) as part of the National Health Mission. Anju challenged the status quo when she decided to freebirth in three subsequent births after having two stillbirths at a public hospital. The rise of freebirthing or “hands off births” has been seen in countries such as Australia, Netherlands, the UK, and the United States, for women's desire to have an intervention free, vaginal, and respectful birth and to understand why from policymakers and how better to meet women's needs.^38^, ^39^, ^40^, ^41^ There are many aspects in the structure of birthing that are violent to women, that violate women.^42^ Women strongly felt against the vaginal examinations when they were given no privacy and were restrained by others, which can be considered a display of physical power. Chawla compares interventions such as routine episiotomy to female genital mutilation, as violence against women who are sexual beings, at their genitals, which is their ‘site of power’.^34^ Regardless of the lack of agency and choice, the gradual process towards taking power away from women is in Indian culture. Respectful maternity care was seen where the family had some influence and cultural capital, such as in case of Pairo where in her second cesarean was much respectful, in her opinion, when compared with her first because her maternal uncle, a doctor himself, had recommended her to her obstetrician. The interplay of these four discourses are essential to understand to ensure respectful care to women.^43^

## Data Availability

The data that support the findings of this study are available on request from the corresponding author. The data are not publicly available due to privacy or ethical restrictions.
